# Pediatric idiopathic anaphylaxis: practical management from infants to adolescents

**DOI:** 10.1186/s13052-024-01712-y

**Published:** 2024-08-09

**Authors:** Francesca Mori, Francesca Saretta, Mattia Giovannini, Mariannita Gelsomino, Lucia Liotti, Simona Barni, Carla Mastrorilli, Luca Pecoraro, Riccardo Castagnoli, Stefania Arasi, Lucia Caminiti, Angela Klain, Michele Miraglia del Giudice, Elio Novembre

**Affiliations:** 1 Allergy Unit, Meyer Children’s Hospital IRCCS, Florence, 50139 Italy; 2 Pediatric Department, General Pediatrician, Azienda Sanitaria Universitaria Friuli Centrale, Udine, 33100 Italy; 3 Department of Health Sciences, University of Florence, Florence, 50139 Italy; 4 Department of Life Sciences and Public Health, Pediatric Allergy Unit, University Foundation Policlinico Gemelli IRCCS Catholic University of the Sacred Heart Rome, Rome, Italy; 5 Department of Mother and Child Health, Pediatric Unit, Salesi Children’s Hospital, Ancona, 60123 Italy; 6 Pediatric and Emergency Department, Pediatric Hospital Giovanni XXIII, AOU Policlinic of Bari, Bari, 70126 Italy; 7 Pediatric Unit, Department of Surgical Sciences, Destiny, Gynecology and Pediatrics, University of Verona, Verona, 37126 Italy; 8 Department of Clinical, Surgical, Diagnostic and Pediatric Sciences, University of Pavia, Pavia, 27100 Italy; 9 Pediatric Clinic, Fondazione IRCCS Policlinico San Matteo, Pavia, 27100 Italy; 10 Division of Allergy, Translational Research in Pediatric Specialties Area, Bambino Gesù Children’s Hospital, IRCCS, Rome, 00165 Italy; 11 Allergy Unit, Department of Pediatrics, AOU Policlinico Gaetano Martino, Messina, 98124 Italy; 12 Department of Woman, Child and General and Specialized Surgery, University of Campania “Luigi Vanvitelli”, Naples, 80138 Italy

**Keywords:** Idiopathic anaphylaxis, Prevention, Diagnostic workup, Adrenaline autoinjectors

## Abstract

Idiopathic anaphylaxis (IA) remains a frustrating challenge for both patients and physicians. The aim of this paper is to focus on IA in pediatric ages and suggest possible diagnostic algorithms according to specific age ranges (infants, children, and adolescents). In fact, in a variable percentage of patients, despite extensive diagnostic tests, the cause of anaphylactic episodes cannot be identified. Moreover, the lack of a unanimous IA definition requires a careful and detailed diagnostic workup. Prompt recognition of signs and symptoms, especially in younger children, and an accurate clinical history often allow a choice of the most appropriate diagnostic tests and a correct differential diagnosis.

## Introduction

In children, “idiopathic anaphylaxis” (IA) is estimated to be present in 10% of all anaphylaxes after a complete allergy workup [1]. However, many differences in epidemiology have been reported by different studies (Table 1).

**Table 1:** Pediatric case series of idiopathic anaphylaxis (IA)

First Author Year, Country	Number of cases Enrollment period	Clinical data Diagnostic work-up (if available)	% IA
Ditto [2] 1997 USA	22 already diagnosed idiopathic anaphylaxis	➔first pediatric case-series median age 10.9 yo 31.8% male 40.9% previous asthma	100%
Calvani [3] 2011 Italy	237 anaphylaxis, 163 enrolled by diagnostic criteria 18 months	➔allergy centers in Italy median age 4.0 yo 68% male 36% previous asthma ➔skin tests, sIgE, tryptase, ProvT	8.4%
Grabenhenrich [4] 2016 Europe Registry	1970 anaphylaxis 7 years + 9 months	➔European Registry 44% < 6 yo, 33% 6–12 yo, 23% 13–17 yo 64.9% male 22.9% previous asthma ➔skin tests, sIgE, tryptase, ProvT	21%
Ganapathy [5] 2016 Singapore	485 anaphylaxis 8 years	➔ED recruitment mean age 8.2 ± 4.3 yo 61.2% male 21.6% previous asthma	33.8%
Wright [6] 2017 USA	40 anaphylaxis 1 year	➔ED recruitment Median age 6.5 yo 70% male 25% previous asthma	17,5%
Lee [7] 2016 Canada	977 anaphylaxis 2 years, 2 centers	➔ED recruitment C1 5.0 yo; C2 6.1 yo C1 male 64.7%, C2 male 56.1% C1 19.7%, C2 18.7% previous asthma	4.9% in C1 10.5% in C2
Le [8] 2019 Canada	295 idiopathic anaphylaxis [3922 total anaphylaxis] 7 years, 8 centers	➔Prospective study 75.3% children < 18 yo, median age 9.0 yo 54.1% male 18.1% previous asthma	7.5% total cases
Gaspar [9] 2021 Portugal Registry	533 anaphylaxis 10 years	➔National Registry Mean age 8.5 ± 4.9 yo 61% male 45% previous asthma ➔IA cases identified though a Registry Questionnaire	1%
Jares [10] 2023 Latin America Registry adults + children	[808 total anaphylaxis] 334 children < 17 yo 42 months	➔international Registry mean age 5 yo 61.4% male 30.8%previous asthma	5.3%

The term IA is usually used to refer to an acute event in which a patient presents the clinical features of anaphylaxis, but no specific cause of anaphylaxis is promptly recognized or identified later and all other diseases that imitate anaphylaxis are excluded along the diagnostic pathway. The term idiopathic anaphylaxis was used for the first time by Bacal et al. in 1978 [11]. One of the first case reports of IA in children was by Dykewicz et al. [12], followed by Ditto et al. [13]. The first pediatric case series on IA was published in 1997 by the same authors’ group [2] and included 22 children, all but one of whom were evaluated at the Division of Allergy and Immunology at Northwestern University (Chicago). These children were previously included in another study from the same group [14] that was later published as a pediatric case series [15]. In the 1997 case series, almost half of the children had other allergic diseases (asthma, rhinitis, food-dependent exercise-induced anaphylaxis, etc.), and 15 out of the 22 (68%) children were female. The children presented a wide variety of phenotypes and erratic responses to therapy. Three children presented with malignant idiopathic anaphylaxis (failure to respond to < 30 mg/day of prednisone or < 60 mg every other day), and one of these was lastly diagnosed with undifferentiated somatoform IA. Some authors suggest investigating this kind of IA when there is no response to any therapy. IA remains a frustrating challenge for both patients and physicians since, in a variable percentage of cases, despite extensive diagnostic tests, the cause of anaphylactic episodes could not be identified. In two very recent reviews on different types of anaphylaxis, the authors [16, 17] reported some possible underlying mechanisms explaining IA; however, most of them are not fully characterized yet. It is of the utmost importance to specify that, over the last few years, some changes in allergy nomenclature regarding IA have been proposed. As pointed out by Hammond [18], signs and symptoms of IA should be considered as a possible presentation of a mast cell disorder, as already proposed by Giannetti et al. [19] and by Akin [20]. Therefore, in managing a patient with a suspected episode of IA, clinicians should always include diagnostic tests for mast cell disorders. Certainly, the lack of a common and precise definition of anaphylaxis introduces further issues to the field. Even the main allergological scientific societies have not yet managed to agree on a unique definition of anaphylaxis (NIAID/FAAN 2006 [21]; EAACI 2020 [22]; EAACI PED [23]; WAO 2020 [24]; ASCIA 2021 [25]; AAAAI 2020 [26]). Furthermore, a uniform definition of AI is also still under discussion. Gulen and Akin [1] compared the typical clinical presentation of IA and mast cell activation syndrome (MCAS), from which it emerges that the involvement of the gastrointestinal system is an exclusive characterization of anaphylaxis if there is a “likely” or “known” cause. In the case of a suspected IA, when the cause remains unknown, cardiovascular symptoms (hypotension, syncope, collapse) or severe respiratory symptoms (laryngeal edema, wheezing, stridor) must accompany skin or mucosal involvement.

Finally, there are no specific diagnostic criteria for anaphylaxis in childhood; therefore, one must rely on adults’ criteria. This is a critical point of discussion since infants and toddlers are especially unable to articulate prodromal symptoms, such as abdominal pain or itching, and fuzziness or sleepiness could be misinterpreted as normal behavior. Of all proposed definitions of anaphylaxis by scientific societies, none provide a dedicated definition for pediatric age. Another critical point is that in most pediatric case series on anaphylaxis (whether enrolled in allergy clinics or in the emergency department [ED]), after a first skimming of food-drug-venom causes, often no further investigation was proposed or allergological referral programmed. Also, as listed in Table 1, not all studies reported a detailed diagnostic pathway through which the IA diagnosis has been formalized. Some studies calculated the percentage of IA only upon the ICD-9 coding at discharge from the ED; others had initially recruited a mixed-case series (adults or children). A clear example of these discrepancies could be observed by comparing the study by Calvani et al. [3], who reported 8.4% IA, and the European registry [4] with 21% IA. Both groups analyzed case series from selected patients already referred to allergy centers, and both had extended diagnostic pathways. In future studies, therefore, it is quite important to include a detailed diagnostic pathway and a long-term follow-up with repeated revaluations. Large studies are needed to define the exact incidence of IA better.

### Pathogenesis

In the recent literature [1, 17], the different pathogenic mechanisms of IA were discussed to a great extent. Gulen T et al. [1] analyzed several features of IA and focused on pathogenesis and the “*intriguing relationship between IA and MCAS and mastocytosis.* Some factors seemed to confirm this connection, such as the main role of mast cells in IA, the commonly released mediators, and the response to therapies targeting mast cells.

In another study by Ivkovic-Jurekovic [27], other mediators seemed to be involved in IA. The authors demonstrated a reduced intestinal or serum activity of diamine oxidase (DAO) and histamine N-methyltransferase (HMT) in three children with IA, confirming their histamine intolerance. The authors also proposed the study of DAO and HMT gene polymorphism as a possible identification of a genetic predisposition but advised that more investigation needed to be done in this regard.

An increase in sensitivity to histamine was proposed by Tedeschi et al. [28]. They demonstrated a positivity of the autologous serum skin test and basophil histamine release assay in a patient as a confirmation of the presence of circulating histamine-releasing factors. However, this theory has not been investigated further.

The presence of autoantibodies against the Immunoglobulin E (IgE) receptor was another hypothesis, but studies did not confirm it [29].

### Diagnosis

The importance of a precise and timely investigation of an anaphylaxis episode was discussed by Gonzales de Olano et al. [16].

Since the authors considered every anaphylaxis as “*the end result of massive mast cell activation*,” they suggested a possible workup, including all diagnostic tests to distinguish different mechanisms of anaphylaxis in the exposures to allergens, exercise, hormones, emotional stress, non-IgE-mediated activation, mastocytosis, and hereditary alpha tryptasemia (HaT).

In this paper, we seek to provide practical information on how to diagnose and manage IA among different age groups (infants, children, and adolescents) and differentiate the possible triggers based on age. Table 2 describes three different scenarios.

**Table 2:** Three different scenarios

Infant	Child	Adolescent
A 3-month female underwent two surgeries with a 2-month interval to correct a cleft lip and palate. No complications occurred during the first surgery; instead, during the second one, she developed a nearly fatal perioperative anaphylaxis	A 5-year-old boy was referred to the Allergy Unit because of the occurrence of urticaria, cough and bronchospasm two hours after dinner. He had eaten pasta with pesto, chicken and grapes. Parents suspected allergy to pine nuts, because usually he eats home-made pesto made with almonds by his grandmother	A 15-year-old boy presented an anaphylactic reaction after eating a piece of nougat: a few minutes after ingestion, the boy presented oropharyngeal itching, angioedema, cough, dysphonia and urticaria. He was treated at ED with intravenous steroids and antihistamines. Signs and symptoms resolved in a few hours. In his past medical history, he had suffered from allergic rhinitis and atopic dermatitis since childhood; in a previous allergic evaluation, he had reported positive skin prick tests (SPT) to Artemisia, Parietaria and grass pollens
Diagnostic work-up and follow up		
After 6 months, she was investigated with in vivo (skin prick tests, intradermal tests) and in vitro (specific IgE, basophil activation) tests for suspected DHR to the drugs administered during surgery—fentanyl, propofol, cefazoline, chlorhexidine, rocuronium—and for latex. All allergy tests resulted negative. A non-IgE mediated reaction was suspected. Acute tryptase dosage was not performed during the acute reaction. Basal tryptase was further measured and high tryptase levels were found (19.1 mcg/ml). The tryptase dosage was then repeated several times with similarly high results (21.2 mcg/ml and 21.3 mcg/ml). Both genetic investigation and bone marrow biopsy confirmed systemic mastocytosis	Prick by prick tests with almond, walnut, hazelnut, pine nut, pistachio, cashew nut and peanuts were negative, as well as skin prick tests with common inhalants extracts (Dermatophagoides pteronissinus and Dermatophagoides farinae, grass, Alternaria, olive, cypress, Parietaria, cat and dog). He was asked to come back with grapes in order to perform prick by prick with fresh fruit, which resulted positive (5 mm), but parents reported that he usually ate grapes without problems, so an in-depth investigation was carried out on what occurred after dinner. His parents remembered the boy was running with his friends. Moreover, parents reported a previous episode of lip angioedema after eating watermelon. At that point a skin prick test was performed with commercial extract of peach Lipid Transfer Protein (LTP) and Pru p3 specific IgE was measured, both with positive results (4 mm and 7.03 KUA/L, respectively). Hence, a food-dependent exercise-induced anaphylaxis (FDEIA) was suspected, and after 2 weeks we performed an oral food challenge with grapes only, which was tolerated. Exercise was then considered as a co-factor in a child primarily allergic to LTP	Skin prick tests with commercial extracts (milk, egg white, tomato, soy, wheat, codfish, cocoa) and with all components of ingested nougat (almond, walnut, hazelnut, peanuts, cocoa and egg white) were performed and all resulted negative. A prick by prick (PbP) with nougat itself also resulted positive. Specific IgEs to such foods were negative, too. Finally, PbP with wildflower honey, contained in the nougat, was positive (4 mm). PbP with chestnut honey, which he had been eating since childhood, resulted 2 mm. Skin prick test and s-IgE to bee, Vespula species, Polistes dominilus and Vespa crabro were negative. Oral food challenge with honey was refused by parents. According with the convincing clinical history and the relevant positive PbP, honey-induced anaphylaxis was diagnosed

Clinical history must be carefully collected, remembering to investigate specific aspects and focus on the role of cofactors (Table 3).

**Table 3:** Clinical History

ANAMNESIS		
Clinical history, e.g.: -patient demographics: age, gender^#^, medical and atopic history, ongoing medications, jobs, hobbies, sports -family history (including unusual reactions/clinical manifestations not otherwise diagnosed) -description of episodes: suspect/known triggers, timing of onset, temperature exposure, time of the day/night, duration, location (e.g. school, home, indoor, outdoor), presence of cofactors* -if further hospital/urgent care access: tests prescribed, therapy needed, response to medications, recovery time or recurrences, need for admission in Pediatric Intensive Care Unit (PICU)		
IF DIAGNOSIS SUGGESTS ALLERGY-DRIVEN ANAPHYLXIS		
Foods	Medications	Sting/bites
-ask for new ingredients (e.g., spices, herbs or foods coloring) -ask for new restaurants/food delivery companies -if fish preparations are a possible trigger: check also for Anisakis (Ani s1) [30] -if wheat is a possible trigger: check for omega-5-gliadin (Tri a19), Tri a14, high-molecular-weight glutenin [31] -if fruit and vegetables are involved: check for oral allergy syndrome to Lipid Transfer Proteins (LTPs), gibberellin-regulated proteins (GRPs), and oleosins proteins [32] -check presence of bee pollens in honey products [33, 34] -check presence of edible insect proteins [35] -ask for consumption of herbal and tea drinks, soft drinks and cocktails [36–38] -if available, read ingredient labels carefully (e.g., synonyms for food allergens) [39] -do not forget of pancakes syndrome [40]	-also consider disinfectants, herbal and alternative products, vitamins, supplements, beauty products as medical products -investigate for possible vaccine allergens: e.g., gelatin, neomycin -although quite rare, Kounis syndrome to drugs or other allergens [41, 42] must be excluded in case of potential clinical manifestations	-although rare, pigeon tick could trigger anaphylactic reactions: the bite of Argas Reflexus, a parasite with nocturnal activity, could elicit allergic reactions including anaphylaxis. In case of skin insect bites, ask about presence of pigeons nearby. Diagnosis could be confirmed with specific IgE determination (Arg r1) [43] -check for insect or animal bites: e.g., Hymenoptera, fire ants, spiders, uncommon insects [44]
Think about allergy to alpha-gal (e.g., mammalian meat or biologic drugs derived from mammalian cell lines), which typically presents as a nocturnal anaphylaxis especially in areas with a high prevalence of tick bites [45]^§^		
Investigate for possible exposure to latex: e.g., hospital/clinic visits, Band-Aid use, toys, balloons, swimming equipment; cross reactivity with fruits (banana, avocado, kiwi, chestnut)		
Aeroallergens: e.g., marijuana		
IF THE CLINICAL SCENARIO SUGGESTS A NON-ALLERGEN DRIVEN ANAPHYLAXIS		
1) exclude mast-cellactivation diseases (MCAD): patients, including children, with systemic mastocytosis (SM) have a higher risk of severe anaphylaxis due to mast cell activation and release of mast cells mediators [46]; therefore, a MCAD must be carefully investigated [47, 48] to exclude SM or other MCAD. Although the most common pediatric presentation of mastocytosis is cutaneous mastocytosis [49, 50], both forms should be investigated, as patients could present systemic signs and symptoms suggesting an anaphylactic reaction 2) exclude HaT: the increase in tryptase should be an indicator to take into account. Be aware that normal tryptase levels vary upon age [51], but a genetic consultation is generally advised if basal tryptase > 8 ng/mL, since more severe reactions are associated with HaT [52, 53] 3) consider other medical diseases with signs and symptoms similar to anaphylaxis such as vancomycin therapy (vancomycin infusion reaction). The latter occurs on exposure to the drug through Mas-related G-protein-coupled receptor member X2 (MRGPRX) activation, pheochromocytoma, carcinoid syndrome, medullary thyroid carcinoma, pancreatic cell tumors [54] 4) consider chronic urticaria/angioedema aggravated by NSAIDs 5) Consider complement activation (CARPA) such as preceding exposure to nanomedicines or biological drugs		

^a﻿^ Pattanaik et al. [55] demonstrated a decrease in IA percentage (from 59 to 35%) after the identification of an alpha-gal allergy

^*^Investigate for presence of extrinsic cofactors as: infections, exercise [consider food dependent-exercise (FDEIA) (e.g., wheat) and exercise induced anaphylaxis (EIA) [56, 57]], alcohol, medications such as nonsteroidal anti-inflammatory drugs (NSAIDs) and proton pump inhibitors (PPIs), psychological stress [58], menses, poor sleep, alcohol, oral mucosal lesions [59]

^#^ In females of fertile age, consider a progesterone hypersensitivity [60]

Other anaphylaxis mimic disorders should be considered in the differential diagnosis of IA, for instance:

- Malignancies such as carcinoid syndrome, VIPoma, familial medullary thyroid carcinoma, and pheochromocytoma
- Bradykinin disorders, hereditary angioedema (HAE), ACE-I (angiotensin-converting enzyme inhibitors) induced angioedema
- Paradoxical vocal cord dysfunction
- Scombroid syndrome
- Vitamin supplements/energy drinks (nicotinic acid)
- Psychiatric diseases, such as Munchausen and Munchausen-by-proxy syndrome [61], undifferentiated somatoform IA [62], and anaphylaxis mimicries, such as psychiatric conditions and panic attacks, should be excluded if diagnostic tests are negative especially if the patient has a history of repeated hospital visits with no firm evidence of anaphylactic reactions.

Based on the possible diagnosis arising from the anamnesis and clinical examination, different types of tests could be performed (Table 4).

**Table 4:** Diagnostic work-up

IN VIVO TESTS
Skin tests for common aeroallergens and food allergens should be performed, especially if IA episodes are reported within about 2 h from meals or after outdoor activities or contact with animals. With new types of foods being launched on the market, such as insect products, carefully tracing the dietary clinical history is mandatory and PbP should be carried out [63] if no extract-based skin prick tests are available. In patients with known pollen allergies, severe forms of oral allergy syndrome should be considered, in particular in those with LTP sensitizations, which could lead to anaphylactic reactions [64]. While considering possible food allergies, it is important to carefully investigate hidden or uncommon allergens, sometimes used as decorations rather than declared ingredients. As pointed out by Bilò et al. [65], failing to identify food allergens usually depends on mislabeling and cross‐contamination
IN VITRO TESTS
**Blood tests** Tryptase The diagnosis can be supported by elevated acute serum tryptase level. Most centers rely on the calculation of a significant increased tryptasemia if the acute total tryptase level is at least 20% plus 2 ng/ml over the patient’s basal tryptase level. However, Mateja et al. [66] evaluated that an acute/baseline tryptase ratio of 1.685 has a sensitivity of 94.4% and a specificity of 94.4% for anaphylaxis diagnosis. Moreover, using a low/high clinical suspicion, the cut-off ratio was 1.868 when suspicion was low and 1.374 when suspicion was high. An online calculator (https://triptase-calculator.niaid.nih.gov) is freely available. Although tryptase levels above the defined normal value (e.g., > 11.4 ng/mL in most laboratories) can be a valuable source of information, some cases of anaphylaxis may not be associated with tryptase elevation. Some authors even argued that certain slight variations could be considered as a normal intra-individual fluctuation. Waters et al. [67] have highlighted the importance of obtaining different baseline tryptase values: among their case-series, the suggested formula (20% plus 2) lacks specificity compared to the above mentioned 1.685 threshold ratio (acute/basal tryptase levels) The correct evaluation of tryptase (at baseline, possibly more than once, and during acute allergic events) [68] has gained more importance in the last years after the definition of HaT [69, 70]. If baseline levels are greater than 8 ng/mL, it is advisable to consider additional tests for HaT. The scientific debate on normal tryptase values is still ongoing, as some authors propose a 1–15 ng/mL normal values interval [71], while other authors argue for individual ranges [72]. Moreover, few specific data on the topic in the pediatric age have been collected so far Specific IgE and Component Resolved Diagnostics Heaps et al. [73] evaluated the use of an allergen microarray (ISAC®, ThermoFisher, Uppsala, Sweden) to gather more information in patients with IA. In 20% of cases, a “highly likely” allergen was identified, previously not detected with skin or specific IgE tests (although no provocation test was performed to confirm a cause/effect relationship). The Component Resolved Diagnostics (CRD) is a useful diagnostic tool both in routine evaluation (e.g., selection of allergen immunotherapy) and in selected cases, e.g. in IA or in patients with multiple allergies or concomitant diseases [74]. Cardona et al. [32] have recently pointed out that, with CRD, a percentage of IA could be resolved, as some of the most important allergens in the field could only be identified with this diagnostic technique (alpha-gal, omega-5-gliadin, lipid transfer proteins and oleosins). Other tests are available in addition to ISAC®, such as Immunolyte® (Siemens Healthcare Diagnostics Inc, Elangen, Germany), Alex2® (MADX, MacroArray Diagnostics GmbH, Wien, Austria), Euroline® (EUROIMMUN AG, Lubeck, Germany) and FABER® (Allergy Data Laboratories srl, Latina, Italy) Basophils Activation Test and Mast Cell Activation Test (MAT) Anaphylaxis could rely on different pathogenesis and it could be completely independent from the classical IgE mediated pathways. Activation and degranulation of MCs and basophils could occur through complement cascade, Mas-related G protein-coupled receptor X2 (MRGPRX2) pathway, or even independently from MCs and basophils [75]. A diagnostic aid could be provided by Basophils Activation Test (BAT) and Mast Cells Activation Test (MAT), unfortunately often available for research purposes only Basophils Activation Test Basophil Activation tests (BAT) could be helpful to confirm CRD or when standard workup turns out negative. Different allergens are available, including perioperative drugs [76] and Hymenoptera venom [77] Mast CellActivation Test (MAT) Another cellular test could be performed, especially when skin tests are not available or indicated [78]. MAT also has some advantages compared to BAT since it does not require fresh samples to be analyzed in a very short timeframe. Moreover, it could be performed with passively sensitized MCs – testing MCs responsiveness beforehand – this way overcoming the non-responders’ issue. MATs could be useful in IgE-mediated and non-IgE mediated reactions with reports such as with aeroallergens, foods and several type of medications [79] ** Other tests** A complete evaluation should always include a panel of the Complement Pathway (C3, C4, C1‐INH functional and quantitative tests, anti-C1-INH antibodies), especially if angioedema is reported 24 h urine collection A 24-h urine collection to include circadian variations should be considered, so as to exclude some anaphylaxis mimicries such as pheochromocytoma, carcinoid syndrome and medullary thyroid carcinoma. Tests may include histamine levels and its metabolites (such as N-methylhistamine or methylimidazole acetic acid), catecholamines and its metabolites (such as dopamine, adrenaline, noradrenaline, vanillylmandelic acid, 5-hydroxyindoleacetic acid), chromogranin A, prostaglandin D2 and leukotriene C4. However, their cutoff levels, specificity, and sensitivities are not well established [54] Evaluation for mast cellactivation diseases A specific mention must be made about the evaluation of MCAD, which includes mastocytosis and MCAS. To formalize a MCAD diagnosis, three diagnostic criteria should be fulfilled: clinical manifestations, MCs activation markers and response to therapy [80]. Once diagnosed, MCAD could be further classified as primary, secondary and idiopathic [81]. Primary MCAD recognizes a clonal origin (such as point mutation D816V in c-KIT and/or aberrant CD25 expression). In secondary MCAS, a non-clonal MCs population is responsible, as MCs are shown to be normal in quantity and function and activated by IgE mediated and non-IgE mediated pathways, such as MRGPRX2 or complement cascade, or physical factors as exercise. In idiopathic MCAS, in which AI has been proposed to be included [19], none of the abovementioned mechanisms could be demonstrated. The importance of a correct and prompt MCAD diagnosis has been addressed by several recent papers [82–85]. Giannetti et al. [86] have also provided specific indications and diagnostic algorithm for the pediatric age Bone marrow biopsy REMA o NICAS score could be used to determine when to perform bone marrow biopsy in patients with recurrent mast cell-mediated symptoms or recurrent IA episodes [48, 87–89]. However, it has been suggested that children, unless demonstrated involvement of spleen, liver, lymph nodes, and peripheral blood are present,do not need to perform a bone marrow biopsy [86, 90]
PROVOCATION TESTS
** Provocation tests** After the identification of a possible specific allergen through clinical history or through a positive allergy test, a provocation test (PTs) could be necessary to confirm the diagnosis. PTs must be performed in hospital settings with specialized personnel and equipment [91]. In case of drug hypersensitivity, if a PT for the culprit drug is not indicated, an alternative drug should be identified [92]. PTs could be also associated with exercise to diagnose FDEIA [93]

**Fig. 1:**
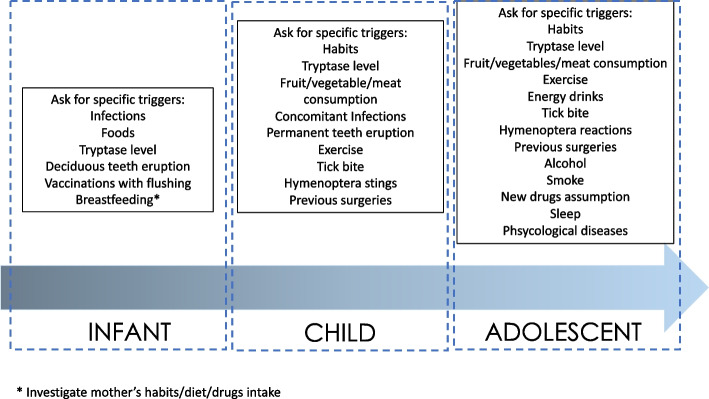
Focused clinical history according to different age ranges

In particular, clinical history should be focused on some peculiarities that differbetween infants, children, and adolescents (Fig. 1). For example, cofactors such as exercise, smoking, alcohol, and psychological distress should not be investigated in infants. On the contrary, other cofactors, such as teeth eruption and vaccinations, should be investigated in infants and children.

Acute management does not differ from classic anaphylaxis. Prompt use of adrenaline is mandatory when cardiovascular and respiratory involvement is recognized. Other medications could be used, such as second-generation antihistamines, steroids, or beta2-agonist inhalers, without delaying adrenaline administration. Education is a cornerstone of the chronic management of IA [54]. If a specific or aggravating factor has been identified and further confirmed, pediatric patients and their caregivers should be instructed on its avoidance, and in the case of food allergens, on reading labels and finding out ingredients. Adrenaline autoinjectors (AAIs) must be provided to patients and/or caregivers as soon as discharged, with a written action plan with detailed instructions on when and how to use AAIs and other drugs (oral second-generation antihistamines, oral steroids, beta2-agonistinhalers). Schools and sports coaches should be involved in AAI use, and formal instruction should be provided. Older pediatric patients should be the direct recipients of education on primary prevention and medical treatment of anaphylaxis. Yearly follow-up evaluations should be scheduled to keep track of IA evolution and to target therapy modifications, if necessary. Patients and caregivers should be advised to avoid all drugs that increase the risk of severe episodes, such as beta-blocking agents, ACE-Is, angiotensin receptor blockers, monoamine oxidase inhibitors (MAOIs), and tricyclic antidepressants (e.g., amitriptyline) [94] If such drugs are necessary, patients should be aware that an impaired effect of adrenaline may occur. As reported by Carter et al. [58], the identification of possible cofactors has a pivotal role in the diagnosis, management, and prevention of further episodes of IA. According to this work, there is also an association between the severity of anaphylaxis and several intrinsic/extrinsic cofactors, age being the most important, followed by concomitant mastocytosis and insects as allergens.

**Fig. 2:**
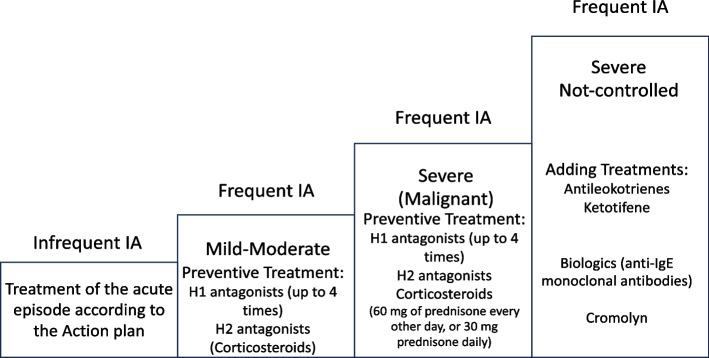
Steps approach of IA treatment

Following diagnosis, it is important to properly classify all patients with IA in order to determine the correct treatment (Fig. 2). IA is commonly categorized as follows [29, 65, 95]:

1. Frequency: infrequent, fewerthan six episodes/year or fewer than two episodes/two months; frequent, more than six episodes/year or more than two episodes/two months;
2. Severity: malignant, patient requires a high dose of steroids for disease control (60 mg of prednisone every other day or 30 mg prednisone daily); corticosteroid-dependent if the IA episodes are difficult to control without steroids;
3. Clinical scenario: generalized with urticaria and/or angioedema and systemic manifestations; angioedema-predominant with angioedema with laryngeal involvement and compromised airway, no other systemic manifestations.

In infrequent IA, there is usually no need for preventive therapy. If the patient is classified with frequent IA, it is reasonable to start a prophylactic therapy, as suggested by many authors, with prednisone and cetirizine daily. H1 receptor antagonists (commonly second-generation antihistamines) could be used with a dose of up to four times per day, as in chronic urticaria. H2 receptor antagonists could also be added to prevent gastric side effects in those who have been treated with oral steroids [54]. In children, particularly those with mild to moderate severity, H1 receptor antagonists and H2 receptor antagonists are preferred [54]. If IA is controlled, a tapering of steroids or an every-other-day scheme could be considered, maintaining daily cetirizine. If IA is not controlled, a step-back to daily prednisone should be made for 1 to 2 weeks [96]. Other suggested therapies, especially in malignant IA or when steroids are needed in high doses, could be adding montelukast (which seems to be effective in children with asthma) and cromolyn in children with gastrointestinal clinical manifestations [54]. Ketotifen could sometimes be helpful in controlling signs and symptoms of urticaria, thanks to its mast cell stabilizer effect [97]. Calcineurin inhibitors and Bruton tyrosine kinase inhibitors are studied in chronic urticaria, but there are no data on their performance on IA. Omalizumab has been proven to be an interesting therapeutic option in IA, generally at the same dosage as in chronic urticaria. Six months could be sufficient as a trial period to assess its efficacy. Kaminsky et al. [98] demonstrated its efficacy in 38 patients (age range 11–54) with IA who failed to respond to second-generation antihistamines and mast cell stabilizers. Of the patients, 63% showed a complete response, 28.5% showed a partial response, and three patients were considered non-responders. There is also additional experience in the pediatric age expressed as case reports but mostly in adolescents [98–103]. Furthermore, there are also a few case reports on the use of dupilumab in IA [104].

Nowadays, the diagnosis and management of IA still remain challenging for clinicians. Prompt recognition of signs and symptoms, especially in younger children, and an accurate clinical history often allow a choice of the most appropriate diagnostic tests and a correct differential diagnosis. It is important, however, to not forget about the rarer conditions that are becoming more frequently diagnosed thanks to the innovations. Over the past decades, the recognition and improvement of knowledge of several novel clinical entities mentioned above have led to a decrease in the percentage of IA, even in the pediatric age group. Nonetheless, further extensive research based on international data is needed, especially regarding those in the infant to adolescent age groups with IA, to improve its management worldwide.

## Data Availability

Not applicable.

## Abbreviations

IA: Idiopathic anaphylaxis
MCAS: Mast cell activation syndrome
NIAID/FAAN: National Institute of Allergy and Infectious Diseases/Food Allergy and Anaphylaxis Network
EAACI: Academy of Allergy and Clinical Immunology
WAO: World Allergy Organization
ASCIA: Australia Society of Clinical Immunology and Allergy
AAAAI: American Academy of Allergy, Asthma & Immunology
ED: Emergency department
DAO: Diamine oxidase
HMT: Histamine N-methyltransferase
HaT: Hereditary alpha tryptasemia
SPT: Skin prick tests
PbP: Prick by test
LTP: Lipid Transfer Protein
FDEIA: Food-dependent, exercise-induced anaphylaxis
MCAD: Mast cell activation diseases
SM: Systemic mastocytosis
MRGPRX: Mas-related G-protein-coupled receptor member X2
NSAIDs: Nonsteroidal anti-inflammatory drugs
CARPA: Consider complement activation
HAE: Hereditary angioedema
ACE-I: Angiotensin-converting enzyme inhibitors
HaT: Hereditary alpha-tryptasemia
CRD: Component Resolved Diagnostics
MAT: Mast Cell Activation Test
BAT: Basophils Activation Test
MCs: Mast cells
PT: Provocation test
AAIs: Adrenaline autoinjectors
MAOIs: Monoamine oxidase inhibitors
